# Genome-wide profiling of 24 hr diel rhythmicity in the water flea, *Daphnia pulex:* network analysis reveals rhythmic gene expression and enhances functional gene annotation

**DOI:** 10.1186/s12864-016-2998-2

**Published:** 2016-08-18

**Authors:** Samuel S. C. Rund, Boyoung Yoo, Camille Alam, Taryn Green, Melissa T. Stephens, Erliang Zeng, Gary F. George, Aaron D. Sheppard, Giles E. Duffield, Tijana Milenković, Michael E. Pfrender

**Affiliations:** 1 Eck Institute for Global Health, University of Notre Dame, Notre Dame, IN 46556 USA; 2 Department of Biological Sciences, University of Notre Dame, Notre Dame, IN 46556 USA; 3 Department of Computer Science and Engineering, University of Notre Dame, Notre Dame, IN 46556 USA; 4 Interdisciplinary Center for Network Science and Applications (iCeNSA), University of Notre Dame, Notre Dame, IN 46556 USA; 5 Notre Dame Genomics and Bioinformatics Core Facility, University of Notre Dame, Notre Dame, IN 46556 USA; 6 Notre Dame Environmental Change Initiative, University of Notre Dame, Notre Dame, IN 46556 USA; 7 Centre for Immunity, Infection and Evolution, Institute of Evolution, University of Edinburgh, Edinburgh, EH9 3FL UK; 8 Institute of Immunology and Infection Research, School of Biological Sciences, University of Edinburgh, Edinburgh, EH9 3FL UK; 9 Present Address: Department of Computer Science, Stanford University, Stanford, CA 94305 USA; 10 Present Address: Department of Biology, University of South Dakota, Vermillion, SD 57069 USA; 11 Present Address: Department of Computer Science, University of South Dakota, Vermillion, SD 57069 USA

**Keywords:** Biological networks, Circadian, Diel, Diel Vertical Migration (DVM), Functional enrichment analysis, Gene expression, Network centrality, Network clustering, Protein function prediction

## Abstract

**Background:**

Marine and freshwater zooplankton exhibit daily rhythmic patterns of behavior and physiology which may be regulated directly by the light:dark (LD) cycle and/or a molecular circadian clock. One of the best-studied zooplankton taxa, the freshwater crustacean *Daphnia,* has a 24 h diel vertical migration (DVM) behavior whereby the organism travels up and down through the water column daily. DVM plays a critical role in resource tracking and the behavioral avoidance of predators and damaging ultraviolet radiation. However, there is little information at the transcriptional level linking the expression patterns of genes to the rhythmic physiology/behavior of *Daphnia*.

**Results:**

Here we analyzed genome-wide temporal transcriptional patterns from *Daphnia pulex* collected over a 44 h time period under a 12:12 LD cycle (diel) conditions using a cosine-fitting algorithm. We used a comprehensive network modeling and analysis approach to identify *novel* co-regulated rhythmic genes that have similar network topological properties and functional annotations as rhythmic genes identified by the cosine-fitting analyses. Furthermore, we used the network approach to predict with high accuracy novel gene-function associations, thus enhancing current functional annotations available for genes in this ecologically relevant model species. Our results reveal that genes in many functional groupings exhibit 24 h rhythms in their expression patterns under diel conditions. We highlight the rhythmic expression of immunity, oxidative detoxification, and sensory process genes. We discuss differences in the chronobiology of *D. pulex* from other well-characterized terrestrial arthropods.

**Conclusions:**

This research adds to a growing body of literature suggesting the genetic mechanisms governing rhythmicity in crustaceans may be divergent from other arthropod lineages including insects. Lastly, these results highlight the power of using a network analysis approach to identify differential gene expression and provide novel functional annotation.

**Electronic supplementary material:**

The online version of this article (doi:10.1186/s12864-016-2998-2) contains supplementary material, which is available to authorized users.

## Background

As organisms progress through their 24 h day, they experience a daily cycle of environmental changes, including rhythms in temperature, light, predation risk, and resource availability. To respond to these daily environmental changes, organisms modulate their biology in a rhythmic manner driven by both the coordinated action of an endogenous circadian pacemaker or clock, as well as the direct effect of the environmental light:dark cycle (LD cycle) [1–8]. True ‘circadian rhythms’ are those rhythms that can be observed under constant conditions, as opposed to ‘diel’ (or diurnal) rhythms that are observed under a LD cycle.

Examples of time-of-day specific biology observed in other organisms include increasing olfactory sensitivity prior to times of foraging [9], rhythmic coordination of detoxification enzymes [10], and time-of-day specific changes in susceptibility to immune challenge [11, 12]. In the disease vector mosquitoes *Anopheles gambiae* and *Aedes aegypti,* gene expression data shows that up to 20 % of the transcriptome is regulated in a circadian and/or diel manner [4, 8, 13]. Similarly, under both circadian and/or diel conditions, as much as 5 % of the transcripts expressed in the fruit fly *Drosophila melanogaster* head and 4 % in the honeybee *Apis mellifera* head are rhythmically expressed [14–16].

**Fig. 1:**
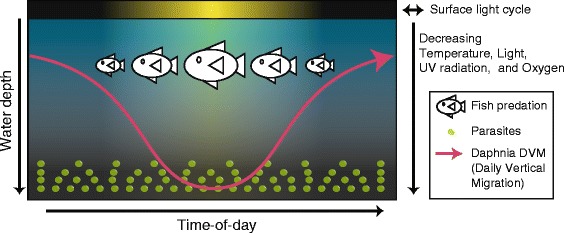
Twenty-four hour rhythmic changes in *Daphnia*’s environment. *Daphnia* are exposed to different environmental conditions and stressors as the 24 h day progresses. This variation is a consequence of the daily rising and setting of the sun, the chronobiology of other organisms in the environment, and *Daphnia*’s pattern of diel vertical migration (DVM) through the water column. The sun brings changes in temperature, ambient light, UV radiation, and increased risk of fish predation (which locate *Daphnia* visually) in populations found in large bodies of water. Similarly, as Daphnids move down the water column they are exposed to decreasing ambient light of changing wavelengths, and reduced UV radiation, temperature, and oxygen levels. Some parasites of *Daphnia* live in the sediment at the bottom of water bodies, so risk of being parasitized is increased at times-of-day the *Daphnia* are lower in the water column (daytime in populations from large water bodies)

*Daphnia* has long been a model for ecological investigation largely because of its cosmopolitan distribution and central role in the trophic cascades of freshwater ecosystems [17]. The addition of genome level information to this well studied ecological model makes it a relevant system for investigating 24 h daily rhythmic biology in a non-insect arthropod [17–19]. Chronobiology research in *Daphnia* has focused primarily on behavioral processes like locomotor activity, daily vertical migration (DVM) through a water column, and mandibular activity (‘feeding’) [20–22]. These phenotypes are all rhythmic under environmental diel conditions. We highlight in Fig. 1 many of the daily environmental rhythms experienced by *Daphnia* species, including temperature, ambient light, and risk of exposure to pathogens and parasites. In *Daphnia*, DVM is thought to be primarily a predator and ultraviolet-radiation (UV-R) avoidance behavior [23, 24]. DVM has been observed in some, but not all reports, to persist even in the absence of a LD cycle [23, 25].

“Preemptive” or “anticipatory” changes in gene regulation and physiology are possible due to the presence of an endogenous cellular molecular circadian clock that provides crucial timing mechanisms in organisms as diverse as cyanobacteria, plants, fungi, insects, and mammals. The circadian clock is cell autonomous and at the molecular level comprises a series of transcriptional-translational feedback loops (TTFLs) whose completion takes approximately 24 h [26]. The genetic underpinning of the metazoan circadian clock is highly conserved [27]. However, even within insects that typically have a conserved genetic construct with regard to their circadian clock, there is some variation in the arrangement of the TTFL components that are found in at least four different functional configurations [28, 29]. As genomic investigations expand into taxonomically diverse arthropods, there is a greater likelihood to find additional variation as well as gain an increased understanding of the genetic basis of rhythmicity.

The investigation of non-model organisms and their genomes is significantly challenged by a lack of functional annotation for many of their protein coding genes. The specific function of many genes, in even well studied organisms, is incomplete. It is not unusual for upward of a third of the genes in any genome to lack functional annotation [30, 31]. This lack of functional annotation is exacerbated in the diverse genomes of many newly sequenced organisms that often contain lineage-specific genes lacking homology with other assembled genomes [19, 31]. The single genome sequence available for the highly diverse lineage of Branchiopod crustaceans is that of the water flea, *Daphnia pulex* [19]. In excess of one third of the *D. pulex* genome comprises lineage-specific genes that lack orthologs in other eukaryote genomes and thus lack any functional annotation [19]. These two factors, diverse clocks and unknown genes, present significant challenges in understanding the rhythmic biology of this species.

Here we describe diel transcriptome analysis performed on female *D. pulex*. Diel as opposed to constant (circadian/ dark:dark) conditions were chosen, as these conditions are more applicable in the context of existing experimental data and indeed natural, real-world environments. This first genome-wide examination of *Daphnia* transcriptional rhythms allows us to explore a wide breadth of crustacean biochemistry, physiology, and behavior that may be under rhythmic control. In addition to an analysis of cyclic behavior using available statistical software (JTK_CYCLE [32]), we employed a comprehensive network modeling and analysis approach to validate and discover both orthologous and novel rhythmic gene regulation in *D. pulex*, as follows.

Established approaches to the statistical analysis of gene expression patterns can provide valuable insights into cellular functioning. However, these analyses often ignore the functional relationships among genes and miss the opportunity to leverage the patterns of co-expression that result from shared regulatory machinery or complex linkage in networks of biological processes [33–36]. For this reason, we modeled *Daphnia* gene expression data as a gene co-expression network, in which nodes correspond to genes and edges link genes with similar expressions [35]. Using the resulting network data, we performed comprehensive computational analyses to search for genes that share topological properties (in the sense that they occupy similar network positions or cluster together) with the rhythmic genes identified by independent analysis of the expression data using JTK_CYCLE. Our goals were to illuminate the transcriptional signature of rhythmicity in a model crustacean and develop a network-based analytical approach to validate rhythmic genes and infer functional relationships for genes lacking functional annotations.

## Results and discussion

### Global transcription analysis and JTK_CYCLE analysis

**Fig. 2:**
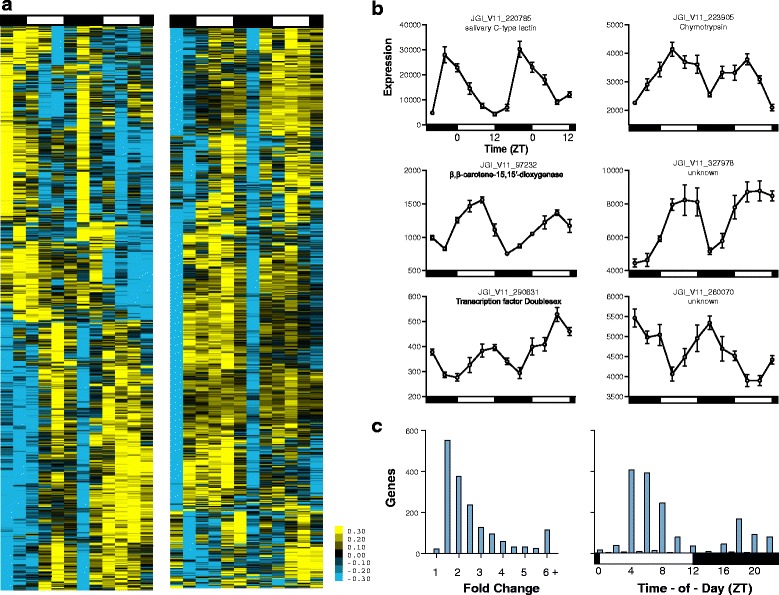
The *Daphnia* genome is expressed in a highly rhythmic manner. **a** Hierarchical clustering of rhythmic *Daphnia* genes. Yellow indicates higher expression, and blue indicates lower expression versus the mean value for each gene. All 1,661 *Daphnia* genes identified as rhythmic by JTK_CYCLE are displayed. The heat map on the right is a continuation of the one on the left. **b** Example gene expression profiles of select genes with low JTK_CYCLE *q*-values. Note the diversity in time-of-peak expression (phase), amplitude of rhythms, and raw-intensity values. Error bars represent S.E.M. of technical replicates. For two of these genes, we performed qRT-PCR confirmation, see Additional file 3. **c** Histograms of rhythmic gene amplitude (peak-to-trough fold change) and times of peak expression. The 1,661 genes called rhythmic by JTK_CYCLE had a median fold change of 2.06. However, there are a significant number of rhythmic genes with a greater than 5-fold amplitude in expression. Peaks of transcriptional expression occur at mid-day and mid-night. Day and night are indicated by the horizontal white/black bars. Histogram X-axis values reflect the minimum fold change/time-of-day reflected in that bin (i.e. >1, ≥1.5, ≥2.0, ≥2.5, etc)

**Fig. 3:**
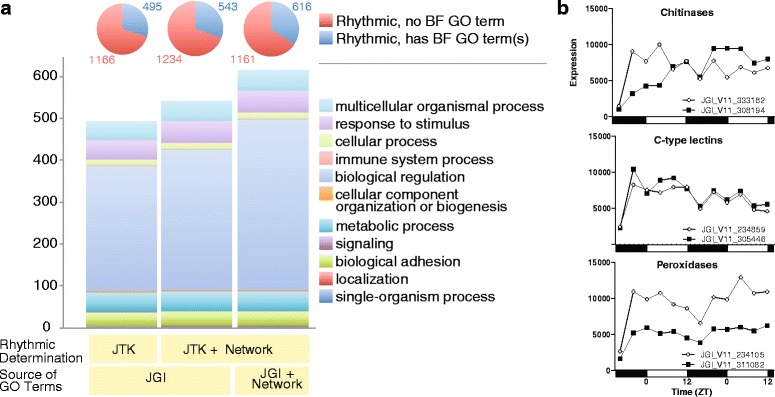
Biological process GO term analysis of JTK_CYCLE and network analysis. **a** Pie charts indicate the number of genes with and without a biological process (“BP”) GO term. Bar charts show the number of genes that have each top level biological process GO term. Genes may have more than one GO term. The three bar charts differ in which rhythmic genes and GO annotations were considered. We used either JTK_CYCLE determined list of rhythmic genes (“JTK”) or the expanded list containing also our network-based rhythmic predictions (“Network”). We used the existing list of GO annotations from the Joint Genome Institute (“JGI”) or the expanded list containing also our network-based predicted GO annotations (“Network”). Whereas this figure displays biological process GO annotations, see Additional file 2 for molecular function GO annotations. Also see Additional file 1. **b** Genes detected as rhythmic by network analysis, but not JTK_CYCLE, that are members of peroxidase, C-type lectins (CTLs), and chitinase gene families described below. See Figs. 6 and 8 for members of the genes families identified as rhythmic by JTK_CYCLE

To perform an analysis of *D. pulex* rhythmic gene expression under laboratory conditions, we profiled genome-wide expression patterns of mature, egg-bearing females maintained on a 12:12 LD cycle with abrupt LD transitions. RNA samples were collected every 4 hours over 2 days and interrogated using custom high-density 12-plex NimbleGen microarrays. Gene expression profiles were mined for 24 h rhythmic, sinusoidal gene expression patterns using the JTK_CYCLE cosine wave-fitting algorithm [32, 37, 38]. From the 21,002 genes with expression levels above background in our microarray experiments, we identified 1,661 genes that were rhythmically expressed using *q* < 0.1 (*p* < 0.03) and 22–26 h period length cutoff criteria (See Fig. 2, Additional file 1). These genes represent 5.7 % of the total *D. pulex* gene set, and 7.9 % of the expressed genes. These rhythmically expressed genes possess diverse biological functions; however, many (14.0 %) of these genes lack any functional annotation (*i.e.*, have no associated orthoDB, KOG, EC, *nor* GO annotation). Visualizations of top-level GO term annotations of the rhythmically expressed genes are provided in Fig. 3a and Additional file 2, and a complete listing is found in Additional file 1. In Fig. 2b we highlight some of the 1,661 rhythmic genes scored with low *q*-values in the JTK_CYCLE analysis; note the diversity in expression in time-of-peak expression (phase), amplitude of rhythms, and raw-intensity values. For two of these highlighted genes, a salivary C-type lectin (JGI_V11_220785) and a β,β-carotene-15,15′-dioxygenase (JGI_V11_97232) we performed microarray validation using qRT-PCR, see Additional file 3.

The medial peak-to-trough fold change of genes determined to be rhythmic by JTK_CYCLE was 2.06. However, many rhythmically expressed genes had much higher daily fold-changes in expression (Fig. 2c). While there were genes with peak expression at all times-of-the day, there were a greater number of genes having a peak expression during mid-day (~ZT 4 – ZT 8) and mid-night (~ZT 16–20) than at other times-of-the day (Fig. 2c). We report peak time-of-day expression in Zeitgeber time (ZT), with ZT 0 defined as the time of lights-on and ZT 12 defined as the time of lights-off.

### Network data

**Fig. 4:**
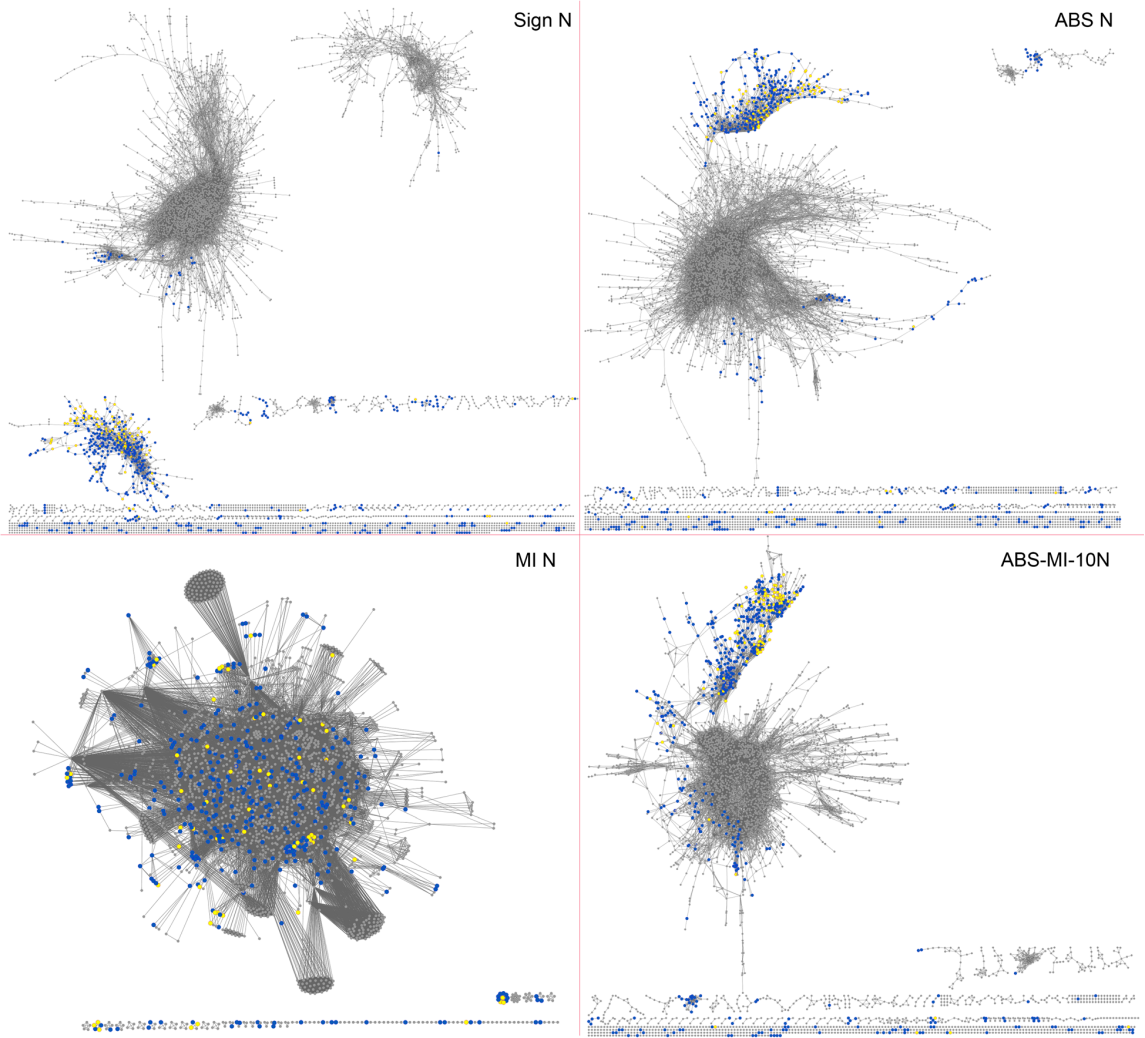
Visualization of the networks from our study. Illustration of SIGN N (signed Pearson correlation with top N interactions), ABS N (absolute Pearson correlation with top N interactions), MI N (mutual information), and ABS-MI-10 N (intersection of absolute Pearson correlation and mutual information with top 10 N interactions) networks. Intersection of absolute Pearson correlation and mutual information with top 10 N interactions is not shown, as it resembles ABS-MI-10 N. In our study, for prediction purposes, we study the networks’ largest connected components. In blue we show the subset of all genes from the given network that are among the 1,661 rhythmic genes determined using JTK_CYCLE statistical analyses of the expression data. In yellow we show the subset of all genes from the given network that are among the *novel* network-based predicted rhythmic genes, *i.e.*, network-based predicted rhythmic genes that could not be identified using the JTK_CYCLE statistical analyses. Note that the differences between some of these networks should not be surprising, since the networks were constructed using different network inference approaches. Also, note that the network visualizations are only intended for illustration. One should not rely on visualizations to determine how meaningful the networks are. For example, what appears to be a group (cluster) of rhythmic genes in the given figure/network might not be reported as a statistically significantly meaningful cluster by network analysis. Or, what appears to be a single cluster in the figure might be broken down and reported as multiple clusters by network analysis. Network analysis (rather than visualization) is a systematic and mathematically/computationally non-ambiguously precise way of interpreting the network data

We modeled the temporal gene expression data with a gene co-expression network as follows: in the network, nodes are genes, and two nodes are connected by an edge if the corresponding genes show similar expression patterns over time. We studied five different networks construction methods using different gene expression “correlation” (*i.e.,* edge weight) measures and different edge cut-offs [35]. These methods included: 1) signed Pearson correlation with top N interactions, 2) absolute Pearson correlation with top N interactions, 3) mutual information with top N interactions, 4) intersection of absolute Pearson correlation and mutual information with top 10 N interactions, and 5) intersection of absolute Pearson correlation and mutual information with top 25 N interactions) (see Methods). Each of these five methods captures a different intuition of how well the genes’ expression levels “correlate” over time. As such, it should be no surprise that some of the networks look different (Fig. 4), have different properties [39] (Additional file 4), and have pairwise intersections that are relatively low (Additional file 5). Because of this, we proceeded by analyzing each of the five networks individually to reveal novel rhythmic genes predicted from their network topology or structure.

### Network-based prediction of rhythmic genes

For each network, we searched in the given network for the subset of the 1,661 genes that have been identified as rhythmic by JTK_CYCLE analysis of the expression data (Additional file 6). Then, we pursued two directions for identifying new rhythmic genes from network topology.

First, we checked if the subset of the 1,661 JTK_CYCLE-identified rhythmic genes that were in the given network were statistically significantly “central” or “peripheral” in the network [36, 40], and if so, we identified other genes with similar network positions and predicted these genes as additional rhythmic genes (see Methods). Here, we used seven popular node centrality, or peripherality, measures, each of which captures somewhat complementary information on the network position of a node [40, 41]: betweenness, closeness, clustering coefficient, degree, eccentricity, graphlet degree, and k-coreness centrality. In this way, we predicted 3,475 genes by at least one combination of network type and centrality measure to be rhythmic, of which 3,019 were not among the 1,661 JTK_CYCLE-identified rhythmic genes and are thus novel. Each prediction was supported by up to 7 combinations of network type and centrality measure (out of the 10 possible combinations; see Methods).

Second, we checked whether the rhythmic genes statistically significantly grouped (*i.e.*, clustered) together in the network, and if so, we found other genes that clustered with them and predicted these genes as additional rhythmic genes (see Methods) [42–44]. Here, we relied on a popular method called Markov clustering algorithm (MCL) [45]. In this way, we predicted 445 genes from at least one network to be rhythmic by the clustering analysis, of which 133 (Additional file 1) were not among the 1,661 JTK_CYCLE-identified rhythmic genes and were thus novel. Each prediction was supported by up to 3 different networks (out of the 3 possible networks, see Methods).

### Validation of the *novel* network-based rhythmic predictions

**Fig. 5:**
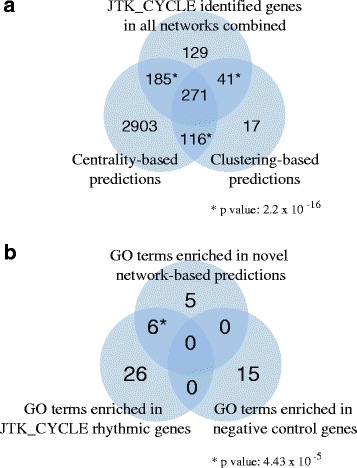
Validation of our network-based rhythmic gene and GO annotation predictions. **a** Pairwise overlaps between the 626 JTK_CYCLE-derived rhythmic genes that are in any of our networks, the network-based rhythmic genes predicted by the centrality analysis, and the network-based rhythmic genes predicted by the clustering analysis. Stars next to the overlap values indicate that all three pairwise overlaps are statistically significant, with *p*-values below 2×10^−16^, which validates our network approach. **b** Validation of our network-based rhythmic genes in terms of GO term overlaps with positive and negative control genes. Pairwise overlaps of enriched GO terms between the JTK_CYCLE-identified rhythmic genes, negative controls, and our novel network-based predictions produced by both centrality analysis and the clustering analysis. Also see Additional file 7

Of the entire set of genes in the expression data, 7,262 genes were present (*i.e.*, non-isolated) in at least one of our five networks (Additional file 6) and could be tested as being rhythmically expressed or not. Of the 1,661 JTK_CYCLE-identified rhythmic genes, 626 genes were present in at least one of our networks (Additional file 6). Recall that we predicted 3,475 and 445 genes by the centrality and clustering analysis, respectively, of which 3,019 and 133, respectively, were not among the 1,661 JTK_CYCLE-identified rhythmic genes and were thus novel (yet, our network-based predictions significantly overlap with the JTK_CYCLE-identified rhythmic genes, with *p*-values below 2×10^−16^, which validates the predictions). Of all predictions, 387 were common to both the centrality and clustering analysis (*p*-value below 2×10^−16^) and were thus of high-confidence, of which 116 were novel (Additional file 1). The overlaps of the different gene sets are illustrated in Fig. 5a.

We provide as comprehensive as possible bioinformatics validation of our *novel* predictions. First, we validated the novel predictions via a functional enrichment analysis. Namely, we split genes from our networks into three sets: 1) the 626 genes identified as rhythmic using JTK_CYCLE that were in any of our networks (as positive control or “known” rhythmic genes), 2) our novel predictions (either the 3019, 133, or 116 predictions from the centrality analysis, the clustering analysis, or both analyses, respectively), and 3) the remaining “negative control” genes that have not been predicted as rhythmic by any approach in our study. Then, we measured the enrichment of Gene Ontology (GO) terms in each of the three data sets, with the hypothesis that our novel network-based predictions would be involved in the same processes (*i.e.*, GO terms) as the JTK_CYCLE-determined rhythmic genes, but not as the negative control genes; and this was exactly what we observed (*p*-value of 4.4×10^−5^ with respect to the hypergeometric test, Fig. 5b, Additional file 7). Second, we further validated our novel predictions by searching for them in an independent set (*i.e*. set not considered when making the predictions in the first place) of “known” rhythmic genes. Specifically, we calculated the significance of the overlap between our novel network-based predictions and sequence homologs of rhythmic [8] mosquito genes as “known” rhythmic genes. Fifty homologs of rhythmic mosquito genes were present in at least one of our networks (anywhere in the given network). Our novel predictions captured a statistically significant portion of these 50 genes (with *p*-values of 0.0028, 0.0129, and 0.0452 for our novel centrality-based, cluster-based, and both centrality- and cluster-based predictions, respectively), unlike the 626 genes identified as rhythmic using JTK_CYCLE (*p*-value of 0.2591) or the negative control genes (*p*-value of 0.999).

These encouraging results (genes in our novel network-based prediction set performing similar functions as, or significantly overlapping with “known” rhythmic genes) imply the effectiveness of the network approach. When combined with the statistical analyses (*i.e.* JTK_CYCLE) of expression data, this approach was able to uncover additional biological knowledge compared to the statistical analyses alone. Of course, in addition to the above bioinformatics validation of our novel predictions, future experimental validation is certainly of interest.

### Network-based prediction of novel functional annotations and their validation

We can use the same network approach as above (*i.e.* the centrality and clustering analyses) to predict novel functional annotations of currently uncharacterized genes based on how well these genes group with functionally annotated genes. In this context, we first validated the accuracy of the network approach by hiding gene-function associations using leave-one-out cross-validation (see Methods) and measuring how accurately we could predict the hidden associations. We found that the prediction accuracy of the network approach is 95 % (*p*-value of 2.2×10^−16^ with respect to the hypergeometric test). Given such a high accuracy, we next applied the network approach to currently uncharacterized genes to predict their functional annotations. In this way, we predicted 477 novel gene-function associations spanning 253 *uncharacterized* genes and 33 GO terms. Also, we predicted an additional 287 novel associations spanning 121 *characterized* genes and 22 GO terms (see Additional file 1). Hence, we demonstrated that our network approach complements significantly and with high accuracy the currently limited functional annotation data (See Fig. 3a, Additional file 1, and Additional file 2.). In the sections below, we highlight genes of interest from various rhythmic gene families. Network analysis revealed *novel* rhythmic genes in only three of these highlighted gene families, peroxidases, C-type lectins, and chitinases. The profiles of these identified genes are visualized in Fig. 3b.

### Rhythmic expression of immune genes

A pattern of diel vertical migration (DVM), which serves to reduce exposure to predators and track resources, is a well-documented behavior in many populations of *Daphnia* living in large bodies of water. This DVM typically manifests as a downward migration during the day and an upward migration at night [23]. While there is a fitness benefit in terms of predator avoidance and resource acquisition, this behavior may also cause increased exposure to pathogens during the daytime since many *Daphnia* parasites tend to live in water body sediments [46]. This observation suggests that there may be an advantage to increasing the transcriptional activity of genes involved in immune processes during the daytime when *Daphnia* are lower in the water column to counteract the increased time-of-day risk of infection.

**Fig. 6:**
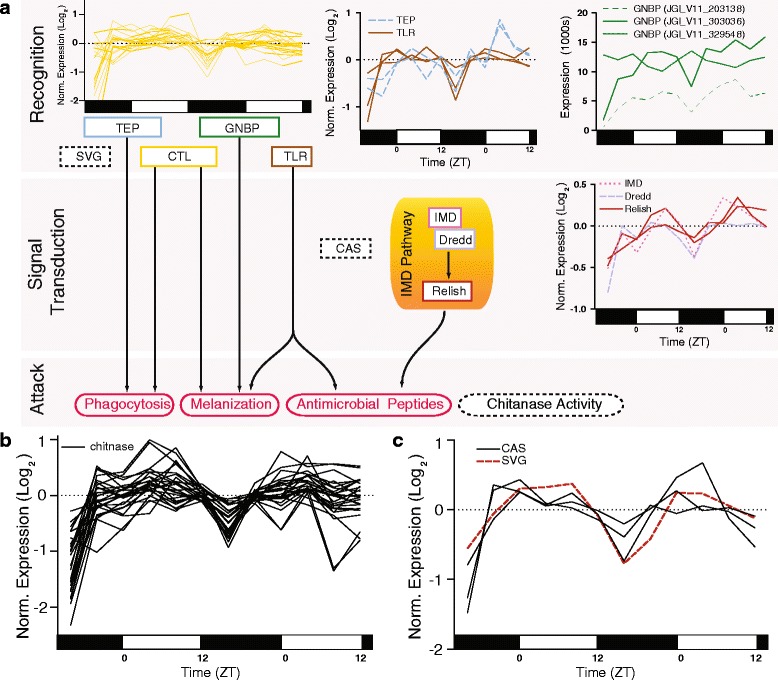
Rhythmic expression of *Daphnia* immune system components. Numerous genes with putative immune functions were found to have rhythmic expression in *D. pulex.* **a** The *Daphnia* immune system can be separated into recognition proteins, signal transduction proteins, and attack proteins and processes. The cartoon illustrates the relationship between these categories. There were a number of rhythmically expressed pathogen recognition genes including two thioester-containing proteins (TEP), nineteen C-type lectins (CTLs), and three gram-negative bacteria-binding proteins (GNBP). Additionally, three membrane-bound toll-like receptors (TLRs) were rhythmically expressed. For signal transduction, four members of the IMD (Immune Deficiency) pathway were also found to be rhythmically expressed: *IMD*, *Dredd*, and two *Relish* paralogs. Expression is presented as raw florescence values for *GNBP*s (for visualization purposes). Two additional rhythmic CTLs were identified using network analysis and these are visualized in Fig. 3b. **b** 32 chitinase (CHT) genes, which may have direct immune functions (as a pathogen-chitinase) were found to have rhythmic expression. For visualization purposes, the 26 with peak phase between ZT4 – ZT6 are presented. Two additional rhythmic chitinases were identified using network analysis and these are visualized in Fig. 3b. **c** A scavenger (SVG) gene and three caspases genes (CAS), which may have immune recognition or signal transduction functions, respectably (but these gene classes also serve other roles), were also found to have rhythmic expression. Day and night for all plots in this figure are indicated by the horizontal white/black bars. Unless otherwise noted, expression in this figure is presented as log_2_ of median normalized expression

Using *D. pulex* immune system genes identified by McTaggart et al*.* [47] and using FlyBase and OrthoDB [48, 49], we identified recognition and signal transduction genes, as well as chitinases that were rhythmically expressed with maximum expression peaking almost exclusively in the daytime (Fig. 6a). There was an abundance of extracellular pathogen recognition molecules [*i.e.,* pattern recognition receptors (PRRs); Fig. 6a; Additional file 1]: First, we identified 19 C-type lectins (CTLs), which activate prophenoloxidase/melanization pathways and promote phagocytosis. Sixteen of these peaked between ZT 4–6 and the other 3 at the very end of night (~ZT 20-0) (Two additional rhythmic CTLs were identified using network analysis and these are visualized in Fig. 3b). Next, three gram negative binding proteins (GNBPs) that activate Toll and prophenoloxidase/melanization pathways were found to be rhythmic (JGI_V11_203138, ZT 6; JGI_V11_303036, ZT 6; JGI_V11_329548, ZT 18). Finally, we identified two rhythmic thioester-containing proteins (TEPs), which promote phagocytosis (JGI_V11_300538, ZT6; JGI_V11_61510, ZT6).

There were three paralogs of Toll-like receptors (TLRs) proteins rhythmically expressed with expression peaking in the morning (JGI_V11_3995, ZT4; JGI_V11_40744, ZT4; JGI_V11_190084, ZT 6; Fig. 6a). These proteins serve as PRRs (but are transmembrane not extracellular proteins) and as components in the Toll signaling pathway. This observation indicates that the Toll pathway may be rhythmically upregulated by means of rhythmic expression of GNBPs and TLR*s*. Similarly, prophenoloxidase activity/melanization may be rhythmic due to the 24 h rhythmic expression of GNBPs and CTLs*.* Note the *GNBP* paralog JGI_V11_329548 had an expression profile that did *not* peak in the morning, but instead in the middle of the night (ZT 18). TEPs and CTLs also promote phagocytosis, so this immune response may also be regulated in a time-of-day specific manner in *D. pulex.*

The IMD signal transduction pathway in *D. pulex* also contained highly rhythmic genes like *IMD* (JGI_V11_313869, ZT 6), *Dredd* (JGI_V11_45861, ZT 4), and two *Relish* homologues (JGI_V11_329057, ZT 6; JGI_V11_62213, ZT 7) (Fig. 6a). This result suggested the IMD immune pathway is more sensitive to activation or will mount a more robust response in the morning. In mice, such a phenomenon exists, with expression of *Toll-like receptor 9* (*TLR9*) and subsequent TLR9 mediated innate and adaptive immunity under control of the circadian clock [50].

Of the 32 chitinases with rhythmic expression, 27 displayed peak expression in the morning (Fig. 6b). Since chitinases can be extracellular they may contribute to rhythmic immunity by directly hydrolyzing the cell walls of chitin-containing pathogens. We note that network analysis revealed an additional two rhythmically expressed chitinases (Fig. 3b).

The rhythmic nature of many immune genes, which generally peak in expression during the daytime (60 of the 69 rhythmic, as determined by JTK_CYCLE, immune genes discussed in this section peak in expression between ZT2 - ZT8), is consistent with the hypothesis that resistance to infection may be higher during the day than the night. Rhythmic levels of extracellular PRRs, chitinases, and IMD signaling pathway components could drive this time-of-day difference. A time-of-day specific resistance to pathogens has been noted in *Drosophila* and mice [11, 12, 50], but remains to be investigated in *Daphnia.*

### Metabolic genes, vesicular-type ATPase subunits, and tRNA synthetases are constitutively expressed

**Fig. 7:**
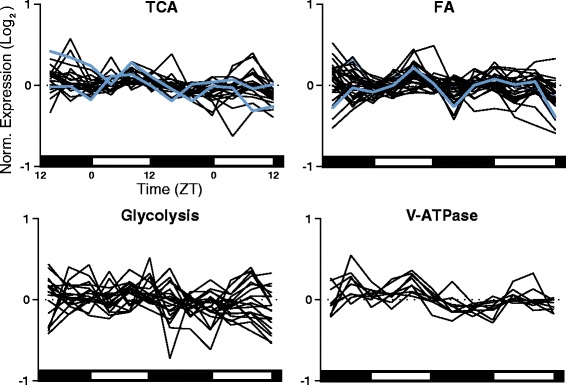
*Daphnia* metabolic processes genes are not rhythmically expressed. Citric acid cycle components (TCA), fatty acid degradation components (FA), glycolysis components, and subunits of vesicular type H+ ATPase (V-ATPase) are almost entirely constitutively expressed over the 24 h day: few genes in these functional pathways were scored as rhythmic by JTK_CYCLE. Exceptions (found rhythmic by JTK_CYCLE *q* < 0.1) are indicated in blue. Network analysis revealed no further rhythmic genes in any of these four gene families. Expression is presented as log_2_ of median normalized expression. Day and night are indicated by the horizontal white/black bars

Organisms over a wide taxonomic span ranging from mosquitoes to mice show extensive rhythmic expression of genes involved in fatty acid (FA) degradation, the citric acid (TCA) cycle, and glycolysis, as well as vesicular-type ATPases (V-ATPase) subunits and aminoacyl-tRNA synthetases [8, 51]. Surprisingly, neither JTK_CYCLE nor network analysis revealed noteworthy rhythmicity of genes involved in these processes in *D. pulex*. Only a few examples of metabolic genes (as identified on the KEGG Pathway Database [52]) were expressed in a rhythmic manner (Fig. 7, Additional file 1). No TCA cycle components were rhythmically expressed despite all annotated components showing transcriptional activity (Fig. 7). Similarly, of the 31 annotated genes playing a role in fatty acid degradation, only a sterol carrier protein X-related thiolase (JGI_V11_221682, ZT 6) and acyl-coA dehydrogenase (JGI_V11_327246, ZT 6) were rhythmically expressed (Fig. 7). Finally, of 20 genes involved in glycolysis, none were rhythmically expressed (Fig. 7). We also do not detect expression rhythms of any *D. pulex* V-ATPase subunits (Fig. 7). In contrast, in *An. gambiae*, heads genes encoding at least 7 enzymes in the glycolysis pathway and 5 in the TCA cycle and 9 of 12 V-ATPase subunits were rhythmically expressed [8].

It is plausible that *D. pulex* primarily regulates metabolic gene expression to match available nutrient reserves and/or ambient temperatures, instead of anticipating future nutrient levels. Under the resource-rich, temperature controlled, and oxygen-stable laboratory conditions, it would therefore not be surprising that we did not detect many rhythmic metabolic genes. Rearing *Daphnia* under cycling temperature conditions or food resource levels in the laboratory will be required to understand if rhythmic metabolic levels indeed exist under cycling environmental condition.

All annotated tRNA synthetases (ligases) displayed constitutive expression (Fig. 7) with the exception of the tRNA synthetase JGI_V11_187913 (ZT 18) (Additional file 1). Rhythms in aminoacyl-tRNA synthetases would suggest an organism has increased protein synthesis activity at particular times-of-day and could indicate there may be rhythmic control at the translational level which produces, enhances, or modifies 24 h rhythms downstream of gene expression. In mosquitoes, 12 of these aminoacyl-tRNA synthetases are rhythmic, and expressed in a similar phase [8], yet in *D. pulex*, we find that expression of annotated tRNA synthetases was primarily constitutive, indicating it is unlikely that there are 24 h rhythms in translation. However, there are various other steps involved in protein translation, post-translational modification, and degradation that could still provide time-of-day regulation and modification of protein levels.

### Sensory processes

**Fig. 8:**
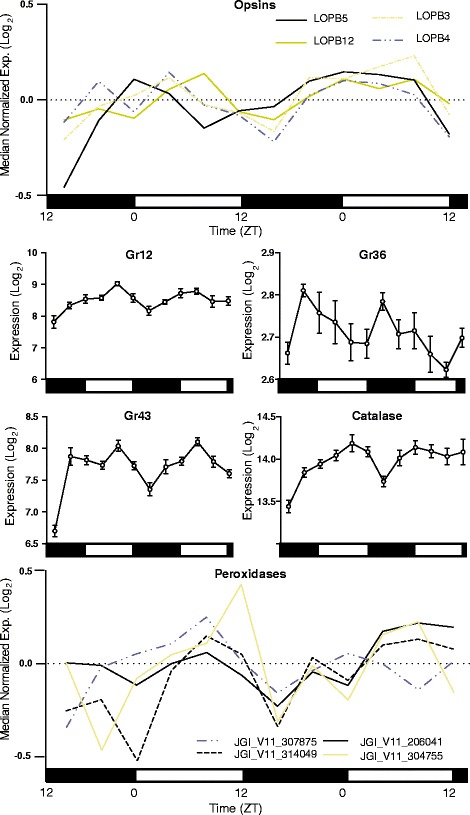
Rhythmic expression of sensory genes and oxidative detoxification genes. Rhythmic expression of LongB clade opsin genes, rhythmic gustatory receptors (Grs), catalase, and rhythmic peroxidases. Expression is presented in log_2_ for single gene plots and log_2_ of median normalized expression for multi-gene plots. Day and night are indicated by the horizontal white/black bars. Error bars are presented for single-gene plots and represent S.E.M. of technical replicates. Additional peroxidases were identified using network analysis and these are visualized in Fig. 3b)

There are a variety of reasons why sensory biology may be modulated by time-of-day. In the mosquito, sensitivity to human host odorants peak at night – the time when the mosquito host-seeks for a blood meal [9]. In a similar manner, *D. pulex* may adjust its sensory processes in anticipation of changing food resource availability or times of greater risk of predation. Electrophysiological studies in numerous terrestrial arthropods reveal sensitivity to light is time-of-day dependent (even under constant conditions, and is thus is not a direct response to changing light conditions). This change in sensitivity compensates for the orders-of-magnitude differences in ambient light changes as the day progresses [53], yet none of the *D. pulex* phototransduction pathway genes identified by Rivera et al*.* [54] were rhythmically expressed in *D. pulex* (Additional file 1). However, we found four rhabdomeric opsins (as annotated by Colbourne et al*.* [19]), all from the LongB clade, were rhythmically expressed (Fig. 8) and with a similar time of peak expression, occurring in the morning (JGI_V11_198385, ZT 4; JGI_V11_305803, ZT 4; JGI_V11_106095, ZT 4; JGI_V11_254506 ZT 6). In addition to changes in ambient light caused by the daily environmental LD cycle, *Daphnia* may respond to changes in light intensity and wavelengths as they move higher and lower in the water column. It has been noted in *Daphnia longispina* that eye pigment migration is under circadian control (observed under both normal LD cycle conditions and constant illumination) [55]. Therefore, instead of regulating the phototransduction cascade to control for changing ambient light conditions, *D. pulex* may ‘shield’ its eyes in a time-of-day specific fashion such has been described in other crustaceans [56].

Daphnids may also adjust their chemoperception abilities/sensitivities in a time-of-day specific manner, for example to respond to changing needs for predator avoidance. Among the gustatory receptors (*Gr*s) identified by Peñalva-Arana et al*.* [57], we found three that were rhythmically expressed (Fig. 8): *Gr12* (JGI_V11_327171) and *Gr43* (JGI_V11_327170), both peaking at ZT 6; and *Gr36* (JGI_V11_329588) which peaks late at night (ZT 22). Gustatory receptors can be very specific in the odorants they detect [58]. It is plausible that *D. pulex* shows time-of-day specific sensitivity to certain odorants, and not others. A similar olfactory phenomena has been observed in the mosquito, which is more sensitive to certain human host odorants during normal host-seeking times-of-day [9]. *D. pulex* could, for example, upregulate its ability to sense predator kairomones at the time-of-day when predation risk is highest.

### Oxidative stress detoxification

It has been reported that *Drosophila* has daily rhythms in resistance to oxidative stress [59]. We examined *D. pulex* genes with known or putative roles in oxidative stress detoxification and found that many were rhythmically expressed (Fig. 8). *Catalase* had rhythmic expression peaking at mid-day (*cat*, JGI_V11_308727, ZT 6; Fig. 8), consistent with the peak time of catalase activity described in *D. longispina* [60]*.* Four genes encoding putative peroxidases (JGI_V11_304755, ZT 8; JGI_V11_307875, ZT 4; JGI_V11_206041, ZT 10; ZT_V11_314049, ZT 10; Fig. 8) were also rhythmically expressed and peak during the daytime (Fig. 8). Interestingly, *sodium oxidase dismutase 1* expression was weakly rhythmic, but with expression peaking much later (*SOD1*, JGI_V11_329848, ZT20; Additional file 1). We note that network analysis revealed an additional two rhythmically expressed peroxidases (Fig. 3b). These data suggest that response to oxidative stress may be time-of-day specific. During the daytime, when sunlight-induced oxidative stress would be most likely to occur, *D. pulex* may be more prepared to respond to oxidative stress.

### Clock genes

We next looked at *D. pulex* homologues of arthropod core molecular clock genes primarily as annotated by Tilden et al. [61]. These ‘canonical’ clock genes make up the transcriptional-translation feedback loops (TTFLs) that encompass the molecular circadian clock. TTFLs have been characterized in a variety of organisms including terrestrial arthropods, rodents, and humans [28, 62]. However, the molecular clock has yet to be characterized in *Daphnia*. Orthologs of the core molecular clock mechanism in *Drosophila* are found in the *D. pulex* genome [61], suggesting that the elements of a conserved arthropod clock are present in *Daphnia*. Tilden et al*.* [61] identified well-characterized circadian clock genes including *clock, cycle*, *period*, *PAR domain protein 1****ε***, *vrille*, eight *timeless* paralogs, and both *cryptochrome 1* (a *Drosophila-*like photoreceptor) and *cryptochrome 2* (a mouse-like transcriptional repressor) [61]. Finding both forms of cryptochrome suggests that the *D. pulex* has an endogenous circadian molecular clock that is more similar to that of the monarch butterfly *Danaus plexippus* and the mosquito *An. gambiae* than to *Drosophila* (which lacks the *cryptochrome 2* transcriptional repressor) [8, 61, 63, 64].

Based on the observation that a full complement of canonical clock gene orthologues were expressed in *D. pulex*, and based on current insect clock models, including those of *Drosophila* and the mosquito, we would predict 24 h sinusoidal rhythmic expression profiles for several of these genes in cells throughout the organism [8, 13–15, 62–66]. This prediction includes at least one, if not both, of the two positive loop components of the TTFL mechanism, *clock* and *cycle*, the three negative loop components, *period*, *timeless* and *cryptochrome 2*, and the members of the interlocking loop, *pdp1* and *vrille*.

**Fig. 9:**
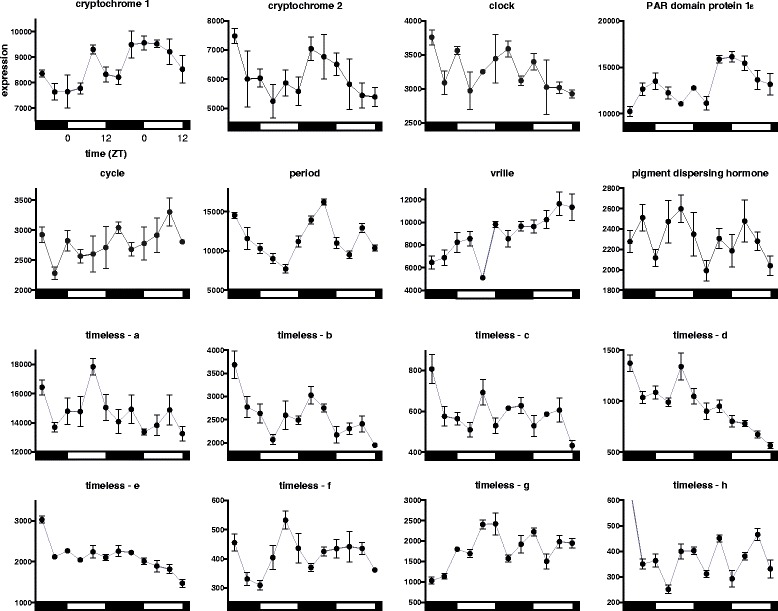
Clock gene expression. Microarray expression of *D. pulex* clock genes. Day and night are indicated by the horizontal white/black bars. Expression is presented as raw florescence values. Error bars represent S.E.M. of technical replicates

Under LD cycle conditions and using our stringent cutoff criteria, our analysis of microarray data did not detect 24 h sinusoidal rhythmic expression of any of the putative canonical clock genes [c*lock, cycle, period, PAR domain protein 1***ε**, *vrille*, *four* of the eight *timeless* paralogs (*a* - *h*), *cryptochrome 1*, *cryptochrome 2*, nor *pigment dispersing hormone* (a neuronal clock output gene, also know as *pigment dispersing factor*)] using JTK_CYCLE or network analysis (Fig. 9, Additional file 1). However, *period* (peak phase ZT 18) and *cryptochrome 2* (ZT 16) were scored as rhythmic by JTK_CYCLE (*q* < 0.05, Fig. 9) when the period length cutoff criteria was relaxed to 28 h, and when the microarray data were subjected to CircWave cosinor analysis (*p* < 0.05; *period*, R^2^ = 0.56, ZT 18; *cryptochrome 2*, R^2^ = 0.55, ZT 17.6) (Fig. 9). Similarly, *timeless-b* (ZT 16)*, timeless-e* (ZT 16), were scored as rhythmic by JTK_CYCLE when the period length was relaxed to 28 h, and *timeless-f* (ZT 8), and *timeless-g* (ZT 8) were rhythmic at a period of 20 h. No significant rhythmicity was detected in any of these *timeless* paralogs by CircWave cosinor analysis. However, there is phase concordance between *period*, *timeless-b*, *timeless-e* and cry*ptochrome 2*, which falls during the early/middle of the night, as is observed in *fruit flies*, mosquitoes, honeybees and some butterflies [4, 8, 13, 14, 29, 64, 66]. This finding would be consistent with the protein products of *period*, *timeless* (specifically *timeless-b* and *timeless-e*) and *cryptochrome 2* functioning as interacting elements of the negative arm of the TTFL mechanism of the molecular clock. Surprisingly, neither *clock* nor *cycle*, the presumed positive loop TTFL components, or *vrille*, exhibit a hint of rhythmicity. While not the focus of this investigation, the lack of a consistent signature of high amplitude, high fidelity rhythmic clock gene expression suggests that the *Daphnia* clock does not exhibit robust rhythmicity in the canonical clock components (transcripts), at least when examined in this case at a whole whole-animal tissue level. Robust rhythmicity has however been described in several other arthropods, including *Drosophila*, mosquitoes, the honeybee and the butterfly [8, 13–15, 64, 66].

There are a number of possible explanations for the lack of robust clock gene rhythmicity detected in this experiment. First, there is evidence that specific tissues of crayfish express clock genes in anti-phase of one another; and in mice the phase of clock gene expression can vary between tissues by as much as 4–8 h [67, 68]. As we assayed entire organisms, it may be that differences in peak phases between tissues reduced the amplitude of the oscillation or appeared as multiple peaks of expression; giving the appearance of a lack of rhythmicity. Second, clock gene rhythmicity may be limited only to a subset of tissues and thus would not be detected in a whole-organism assay. For example, rhythmic *clock* gene expression was not detected in all peripheral tissues of the Zebrafish [69].

It is also feasible that *D. pulex* has an alternative, non-canonical, core molecular clock that operates differently from well-characterized insect clocks. In fact, under constant light conditions, *D. magna* has been reported to have an unusually long 28 h free running period [23, 70]. Alternate clock mechanisms have been suggested in a number of invertebrates including the nematode, *Caenorhabditis elegans* [71], the sea squirt, *Ciona intestinalis* [72], and in other crustaceans like the speckled sea louse, *Eurydice pulchra* [73], and the prawn, *Macrobrachium rosenbergii* [74]*.*

In summary, under our laboratory diel conditions and while assaying whole organism transcriptomic data, robust, high fidelity, high amplitude, 24 h sinusoidal expression of *Daphnia* canonical clock genes was not detected. We suggest several possible explanations, but we cannot distinguish among these alternatives. Future experiments to determine the mechanism of *Daphnia* circadian clocks should examine tissue-specific expression patterns of clock genes under both diel and constant dark conditions.

### Comparison with condition-dependent gene regulation

We next explored the overlap between *D. pulex* genes we identified as rhythmic and those previously identified as having differential expression under various experimental manipulations. Table 1 highlights the proportion of genes previously identified as differentially expressed (q < 0.05) under differing environmental temperatures [75], salinities [76], and resource manipulations of carbon: phosphorus ratio [77] that we also found to be rhythmic (JTK_CYCLE *q* < 0.05). For example, 38 % of genes differentially regulated in response to salinity stress were also found to be rhythmically expressed. The degree of overlap suggests that not controlling for RNA collection time across treatments or replicates could result in false positives or false negatives in differential expression studies. Future experimental designs should take these findings into consideration and time-of-day specific effects should also be considered when interpreting previously collected data.

**Table 1:** Comparisons between condition-dependent and temporal regulation of gene expression

Experiment	Conditions	Genotypes	Overlap ^d^
Thermal regime ^a^	18 °C vs. 28 °C	Low tolerance genotype C	11 % / 183
		Low tolerance genotype E	12 % / 199
		High tolerance genotype B	7 % / 116
		High tolerance genotype K	7 % / 116
Salinity stress ^b^	5 gL^−1^ NaCl vs. control	High tolerance genotype	38 % / 631
		Low tolerance genotype	33 % / 548
Carbon:Phosphorous ratio ^c^	HiC:LoP (C:P ~800) vs. LoC:HiP (C:P ~100) (3 days)	G1: LoC:HiP tolerance	4 % / 66
		G2: HiC:LoP tolerance	8 % / 133
	HiC:LoP (C:P ~800) vs. LoC:HiP (C:P ~100) (6 h)	G1: LoC:HiP tolerance	16 % / 266
		G2: HiC:LoP tolerance	9 % / 149

^a^Yampolsky et al. 2014 [75]

^b^Latta et al. 2012 [76]

^c^Chowdhury et al. 2014 [77]

^d^The number and percent of genes both differentially expressed in the given experimental treatment conditions (*q* < 0.05) and also rhythmically expressed as determined by JTK_CYCLE in this present study (*q* < 0.05)

## Conclusions

We present genome-wide diel transcriptional profiling of *Daphnia* under controlled laboratory conditions. Our analysis revealed that *Daphnia* express a great number and diversity of genes in a highly rhythmic manner, including genes involved in sensory processes, response to oxidative stress, and immune-related genes. It is advantageous for an organism to upregulate its ability to combat or avoid environmental stressors at the times-of-day they are most likely to encounter such risks. We highlighted how during the daytime, *Daphnia* populations may be at greater risk of oxidative damage (from the Sun), predation (from fish, which locate them visually during the day), and exposure to pathogens (which they encounter at greater frequency at the bottom of the water column in Daphnia populations that migrate downwards during the day-time). It therefore should not be surprising that genes that function in detoxifying oxidative stress, chemoperception, and combating pathogens are rhythmically expressed.

We showed that while *D. pulex* has a full complement of the core clock genes found in other animals, the expected high amplitude rhythmic expression of these genes is not apparent when examined at the whole animal level. Work in other crustaceans suggests that clock rhythmicity may be limited to a subset of tissues or has anti-phasic expression in various tissues that would obfuscate the expression patterns of these genes in a whole-organism assay. It is also possible that *D. pulex* has an alternative molecular clock. Our work contributes to a growing understanding that there is less conservation than initially reported in the mechanisms of arthropod clocks and that more investigation is prudent in elucidating the core clock of *Daphnia*.

Finally, we developed a comprehensive network modeling and analysis approach that complements and enhances the standard statistical analyses of differential expression (*e.g.,* JTK_CYCLE). This network analysis, when built on top of the statistical analyses, revealed additional knowledge about rhythmicity that could not be captured by the statistical analyses alone. Importantly, we demonstrated the usefulness of the network approach to identify novel functional annotations for currently uncharacterized genes. As the number of available arthropod genome sequence assemblies increases, and the taxonomic breadth of these genomes widens, methodological approaches that take into account the patterns of co-expression and network configuration will become vital additions to the process of functional annotation.

Future work may include examining rhythms at the protein and functional level and examining rhythms in specific tissues (especially in regards to clock gene expression). Further, because other Zeitgebers (time-givers), such as temperature rhythms, may enhance observed rhythms [78], their role in governing *Daphnia* rhythmicity should also be investigated.

## Methods

### Experimental material, RNA collection, extraction, and microarray hybridization

A *D. pulex* genotype was collected from a vernal pond in southwestern Michigan (42.31979 N 85.35837 W) on May 1, 2011. This pond, referred to as “Roughwood”, has a maximum depth of <2.0 M. Single adult females were isolated in the lab to initiate isoclonal cultures and these lines were maintained through parthenogenetic reproduction. The experimental time course was performed in August of 2011. Stock cultures of this genotype (Roughwood 40-11) were maintained for three generations at 18 **°**C in 1 quart jars containing COMBO water medium [79] and a 12 L:12D photoperiod (with abrupt transitions). The media, containing resource in the form of algae *Scenedesmus acutus* culture at a concentration of 200,000 cells/ml, was replaced every second day to maintain a constant level of resource and individuals were culled to maintain a density of 50 individuals per culture. To establish the experimental generation, female offspring from the second or third clutch of second-generation females were collected within 48 h of birth, pooled, and divided into twelve 1 quart jars containing 750 ml of COMBO media and algae and maintained as above with the same feeding regime and 12 L:12D photoperiod. When these third generation females were 10–12 day old the experiment began and replicate pools of 30 animals per jar were collected, rinsed in distilled water, and snap frozen on dry ice every 4 h over a 44 h period.

To assess patterns of gene expression, we used the *D. pulex* Expression Array 12x135k GEO Accession GPL11278 (See Colbourne et al*.* [19]). The platform is a high-density NimbleGen (Roche-NimbleGen, Madison, WI) microarray that accommodates 12 experiments per glass slide, with each experiment interrogating 137,000 probes. Each predicted and experimentally validated gene is represented by as many as three probes, whereas the remaining probes are designed from transcriptionally active regions whose gene models are not yet known.

Total RNA was purified from a single pool of animals collected at each time point using TRIzol reagent (Invitrogen, Carlsbad, CA) extraction followed by RNA purification using a Qiagen RNeasy Mini Kit with on-column DNAse treatment to isolate total RNA. RNAsecure (Ambion, Austin, TX) was used after RNA purification to inactivate any remaining RNAases. The quality and quantity of resulting RNA were assessed using a Nano Drop (Thermo Scientific, Waltham, MA) and RNA Nano LabChip for the Bioanalyzer (Agilent Technologies, Santa Clara, CA). Beginning with 1.0 μg of total RNA, a single round of amplification using MessageAmpTM II aRNA kit (Ambion) was performed for each RNA sample. The cRNA (10 μg) was converted to double strand cDNA with random primers using the Invitrogen SuperScript Double-Stranded cDNA Synthesis Kit (Invitrogen). From 1 μg double-stranded cDNA, labeled cDNA was generated with NimbleGen’s Dual-colour Labeling Kit (Roche NimbleGen).

RNA from each pooled sample was used to create four technical hybridization replicates. Dual-colour hybridization, post-hybridization washing, and scanning were done according to Roche NimbleGen protocols [80]. Images were acquired using a NimbleGen MS 200 with 2 μm resolution, and GenePix 6.0 software (Molecular Devices, Sunnyvale, CA). The image data from these arrays were processed using NimbleScan v2.5 software (Roche NimbleGen) to extract probe intensity values. These data have been deposited in the NCBI Gene Expression Ominibus and are accessible through GEO accession number GSE67781.

### Microarray data analysis

Gene expression values (*i.e.,* gene intensity values) were obtained from a summarization of intensity values of all corresponding probes using the RMA (Robust Multi-array Average) method [81]. The pre-processed microarray data were imported into an in-house analysis pipeline using Bioconductor [82] for normalization and analysis. All genes were quantile-normalized across arrays, samples, and technical replicates [83]. Unless otherwise noted, data presented is normalized, but not log transformed.

### JTK_CYCLE analysis of rhythmic gene expression

To determine the patterns of rhythmic gene regulation we first used the JTK_CYCLE algorithm as previously described [32, 37, 38]. JTK_CYCLE is a nonparametric statistical algorithm designed to identify and characterize cycling variables in large datasets. It applies the Jonckheere-Terpstra-Kendall (JT) test and Kendall’s tau (rank correlation), to find the optimal combination of period and phase that minimize the *p*-value of Kendall’s tau correction between the experimental time series and each tested cyclical ordering, this being derived from cosine curves. JTK_CYCLE generates period length, phase, and amplitude estimates, as well as corrects for multiple comparisons *post hoc*. The reported *q*-value takes into consideration the false discovery rate (FDR) across all genes [84].

Prior to running JTK_CYCLE, genome features on the microarray not mapping to a gene in the *D. pulex* v1.1 frozen gene set were excluded, as were all probes that failed to exceed background levels in at least one sample. A background fluorescence cutoff value of 136.5 was used as it was a value that excluded >99.8 % of random probes on the arrays. Genes were scored as being rhythmic if they were found using the JTK_CYCLE algorithm to have a *q* < 0.1 (a commonly used JTK_CYCLE cutoff) and period length of 22–26 h.

Gene annotations followed the 2013–2014 gene annotations from the Department of Energy – Joint Genome Institute *Daphnia* Genome Browser [85] supported by gene annotations from OrthoDb [48] and euGenes Arthropod 2009.12 annotations [86]. Further gene and gene family identifications were taken from Rivera et al. [54], Colbourne et al. [19], McTaggart et al. [47], Peñalva-Arana et al. [57], KEGG Pathways Database [52], and from 2013 to 2014 *Drosophila melanogaster* homologs listed on FlyBase [49]. For each gene family mentioned in this paper (e.g., chitinases, putative clock genes, glycolysis, etc.), the identities of all genes considered for that family are provided in Additional file 1.

### Hierarchical cluster analysis

We performed hierarchical cluster analysis (Fig. 2) using Cluster 3.0 and visualized the results using Java TreeView. Data were mean centered, normalized across the time course for each gene, and centroid linkage clustering performed [87, 88].

### Real-time quantitative RT-PCR (qRT-PCR) analysis

Total RNA (as above) was used for cDNA synthesis using a High Capacity cDNA reverse transcriptase kit (Applied Biosystems, Foster City, CA) primed with random hexamers. PCR thermocycling and qRT-PCR were performed using SYBR green reagents per manufacture’s protocol using an Applied Biosystems 7500 Fast Real-Time PCR System. Quantification was based on the generation of standard curves. Dissociation curves to test for primer dimers were generated using dissociation curve software (Applied Biosystems). Normalization of genes was calculated relative to a *D. pulex* gene deemed constitutively expressed over the 24 h, *Alpha tubulin* (JGI_V11_301837). See Additional file 3 for primer sequences used.

### Cosinor analysis

CircWave v1.4 software, an extension of cosinor analysis, was also used to analyze clock gene expression rhythmicity. This analysis fits a Fourier curve (one sine wave) to the data. The *p* values reported are the result of *F*-tests from the software [89]*.*

### Network construction

We modeled temporal gene expression data with a gene co-expression network as follows: in the network, nodes are genes, and two nodes are connected by an edge if the corresponding genes show similar expression patterns over time. To measure similarity between expression profiles of two genes, we used one of the following three popular edge weight methods: signed Pearson correlation, absolute Pearson correlation, and mutual information, see Rider et al*.* [35] for details. Each edge weight method assigned to every pair of genes a score that captures some intuition of how well the genes’ expression levels “correlate” over time; then, we constructed a co-expression network by predicting the top *K* highest-scoring node pairs as edges in the network. We varied *K* from *N* (where *N* is the number of genes in the expression data) to 10 *N* in increments of *N*, and we also tested *K* = 25 *N*, 50 *N*, and 100 *N*, in order to evaluate the effect of the value of parameter *K* on the resulting network structure. Intuitively, we wanted to choose this value in a way that keeps only significant edges and provides a meaningful representation as well as interpretation of the data [90]. Namely, for each edge weight method, we aimed to construct a network that ideally linked all genes (*i.e.*, has no isolated nodes), in order to include into the network as much information from the data as possible. At the same time, we aimed to construct a network that is not too dense (where density is defined as the number of edges in the network out of all possible edges), in order to mimic the sparse nature of many real-world networks as well as avoid randomness in network topology [90, 91]. Empirically, by studying the number of non-isolated nodes, edges, connected components, and nodes and edges in the largest connected component, we found that as *K* increases, the number of non-isolated nodes barely increases, while the network density increases drastically. Therefore, since larger values of *K* increase network density without introducing many new nodes into the networks, which could unnecessarily increase computation complexity of network analysis methods or include “lower-confidence” interactions, we empirically decided to focus on the following networks (*i.e.,* their largest connected components): signed Pearson correlation with top N interactions, absolute Pearson correlation with top N interactions, and mutual information with top N interactions. Also, since edges that are captured by both Pearson correlation as well as mutual information could be of higher confidence, we also studied (largest connected components of) the intersections of absolute Pearson correlation and mutual information with: 1) top 10 N interactions and 2) top 25 N interactions. Thus, we studied a total of five networks.

### Network-based prediction of new rhythmic genes – node centrality-based analysis

For a given network and a given centrality measure, *i.e*., for each of 5×7 = 35 possible cases (where 5 corresponds to the number of studied networks and 7 corresponds to the number of studied centralities), in 11 cases we observed statistically significant differences (*p*-values below 0.01) between network positions of the rhythmic genes and the remaining genes. Next, for each of the 11 cases, we ranked all genes in the network from the most central to the least central (or vice versa). We took the top *K*% most (or least) central genes, where we varied *K* from 1 to 100 % in increments of 1 %. Then, we computed precision (the portion of the top *K*% genes that are JTK_CYCLE-identified rhythmic genes), recall (the portion of JTK_CYCLE-identified rhythmic genes from the given network that are among the top K% genes), and F-score (which combines precision and recall into a single value that is easier to interpret than the two individual and typically “contradictory” measures). At the value of *K* where F-score peaks (meaning that the methodology achieves the highest prediction accuracy at that value of *K*), we measured the enrichment of the top *K*% genes in the JTK_CYCLE-identified rhythmic genes (with respect to the hypergeometric test). In 10 out of the 35 possible cases, we observed statistically significant enrichments signal (with *p*-values below 0.05 after Bonferroni correction for multiple hypothesis testing, which was done using p.adjust package in R). In each of the 10 cases, we took the remaining (non-JTK_CYCLE-identified) genes among the top (or bottom) K% genes and predicted them as network-based rhythmic candidates. We recorded how many of the 10 combinations of network type and centrality measure support each prediction, because the more the combinations, the higher the prediction confidence.

### Network-based prediction of new rhythmic genes – network clustering-based analysis

We clustered each network with Markov clustering algorithm (MCL) (with the inflation parameter set to 2, because this value gives empirically optimal cluster size distribution, *i.e.*, not too many very small clusters or too few very large clusters). Then, focusing on meaningful clusters (of size at least two and containing at least two JTK_CYCLE-identified rhythmic genes), for each value of *K* in 1–100 % range, we found all clusters that have enrichment in the JTK_CYCLE-identified rhythmic genes greater than *K*% (*i.e.*, clusters in which at least *K*% of the genes are JTK_CYCLE-identified rhythmic genes), and we measured the statistical significance of the enrichment via the hypergeometric test (at a *p*-value threshold of 0.05 after Bonferroni correction for multiple hypothesis testing). For each of the resulting significant clusters, we predicted the genes in the cluster as network-based rhythmic genes. We took all predictions from all significant clusters and computed the overall prediction accuracy via precision, recall, and F-score measures. Further, in order to ensure that we could not achieve the same prediction accuracy by chance, we randomly clustered the network 100 times and repeated the above steps to compute “randomized” precision, recall, and F-score. Then, we used these randomized results to determine “optimal” K at which to make predictions from the real clusters. Namely, we selected the value of *K* at which F-score peaks, but only if that F-score value is statistically significantly high compared to the randomized F-score values. For three of our five networks (absolute Pearson correlation with top N interactions, intersection of absolute Pearson correlation and mutual information with top 10 N interactions, and intersection of absolute Pearson correlation and mutual information with top 25 N interactions), results are statistically significantly better from the actual clusters than from the randomized clusters, and we predicted new rhythmic genes from these three networks (and we left out the other two networks from consideration when making predictions). We recorded how many of the three networks support each prediction, as the higher the number of networks, the higher the prediction confidence.

### Network-based prediction of novel functional annotations and their validation

To validate the network approach in the context of predicting novel gene-function associations, we used leave-one-out cross-validation, as follows. When considering all genes in any one network, we hid functional information for one gene of interest at a time. Then, we used functional information about all other genes that group with the gene of interest (with respect to either centrality of clustering analysis) to potentially predict function(s) of the gene of interest. Namely, if the given gene group is statistically significantly enriched in a given function (with *p*-value below 0.05 after Bonferroni correction for multiple hypothesis testing), we annotated the entire gene group with that function, including our gene of interest. We repeated this procedure for all genes and got the set of predicted gene-function associations for each of centrality and clustering analysis. For increased confidence, we considered association predictions that are in the intersection of the two analyses. For the resulting predictions, we measured their accuracy via precision, which is the percentage of associations that we predicted via the network approach which are in the known functional annotation data. Novel predictions are provided in Additional file 1.

## Abbreviations

CHT, chitinase; CTL, C-type lectin; DVM, diel vertical migration; FA, fatty acid; GNBP, gram-negative bacteria-binding protein; GO, gene ontology; Gr, gustatory receptor; IMD, immune deficiency; LD, light:dark; MCL, Markov clustering algorithm; PRR, pattern recognition receptor; SIGN N, signed pearson correlation with top N interactions; TCA the citric acid; TEP, thioester-containing protein; TLR, toll-like receptor; TTFL, transcriptional-translational feedback loop; V-ATPase, vesicular-type ATPase

## Additional files

Additional file 1: JTK_CYCLE and network analysis results. The spreadsheet shows, for each *D. pulex* gene: 1) Gene annotations 2) JTK_CYCLE derived statistics, 3) network analysis rhythmic gene predictions and statistics, and 4) network analysis functional annotations, predictions, and statistics. (XLSX 1853 kb) Additional file 2: Molecular function GO annotations of our rhythmic genes. Pie charts indicate the number of genes with and without a molecular function GO term. Bar charts show the number of genes that have each top level molecular function GO term. Genes may have more than one GO term. The three bar charts differ in which rhythmic genes and GO annotations were considered. We used either JTK_CYCLE determined list of rhythmic genes (“JTK”) or the expanded list containing also our network-based rhythmic predictions (“Network”). We used the existing list of GO annotations from the Joint Genome Institute (“JGI”) or the expanded list containing also our network-based predicted GO annotations (“Network”). Whereas this figure displays molecular function GO annotations, see Fig. 3 for biological process GO annotations. Also see Additional file 1. (TIF 2814 kb) Additional file 3: Microarray validation using qRT-PCR. Microarray validation was performed on two rhythmic genes, a salivary C-type lectin and a β,β-carotene-15,15′-dioxygenase using qRT-PCR. The qRT-PCR gene expression is relative to *Alpha tubulin*. Day and night are indicated by the horizontal white/black bars. Primer sequences are provided. (PDF 271 kb) Additional file 4: Properties of our networks. The size, density, average diameter, and average clustering coefficient of the five networks (*i.e.,* their largest connected components), which we consider in our study. We also studied additional network properties, including the degree distribution, clustering spectrum, and graphlet frequencies (results not shown). (DOCX 57 kb) Additional file 5: Intersections between our networks. Pairwise network intersections, in terms of the number of edges common to two given networks divided by the number of edges present in the union of the two networks. (DOCX 55 kb) Additional file 6: Information on the rhythmic genes in our networks. The number of all nodes or the subset of the 1,661 JTK_CYCLE-identified rhythmic genes, for each network and all five networks combined. (DOCX 68 kb) Additional file 7: Validation of our network-based rhythmic genes in terms of GO term overlaps with positive and negative control genes. Pairwise overlaps of enriched GO terms between the JTK_CYCLE-identified rhythmic genes, negative controls, and our novel predictions produced by (A) centrality analysis or (B) the clustering analysis. (PDF 347 kb)
