# Supplementary figures and images for: Genome-wide profiling of 24 hr diel rhythmicity in the water flea, Daphnia pulex: network analysis reveals rhythmic gene expression and enhances functional gene annotation
Source: BMC Genomics. 2016 Aug 18;17:653. doi: 10.1186/s12864-016-2998-2 (PMC4991082; doi:10.1186/s12864-016-2998-2)

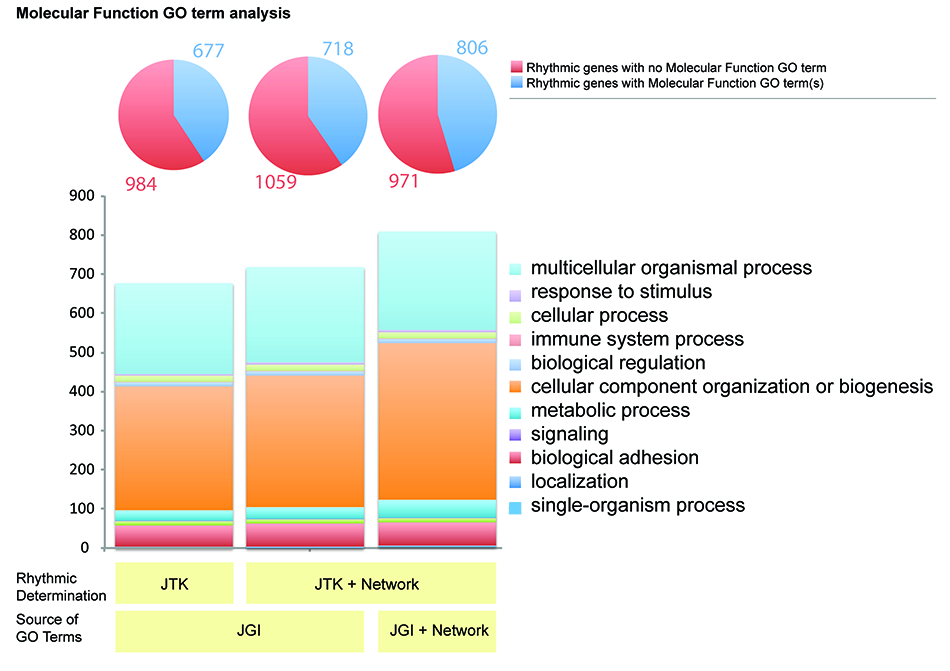

Supplement: Additional file 2: — Molecular function GO annotations of our rhythmic genes. Pie charts indicate the number of genes with and without a molecular function GO term. Bar charts show the number of genes that have each top level molecular function GO term. Genes may have more than one GO term. The three bar charts differ in which rhythmic genes and GO annotations were considered. We used either JTK_CYCLE determined list of rhythmic genes (“JTK”) or the expanded list containing also our network-based rhythmic predictions (“Network”). We used the existing list of GO annotations from the Joint Genome Institute (“JGI”) or the expanded list containing also our network-based predicted GO annotations (“Network”). Whereas this figure displays molecular function GO annotations, see Fig. 3 for biological process GO annotations. Also see Additional file 1. (TIF 2814 kb) [file 12864_2016_2998_MOESM2_ESM.tif]

A.

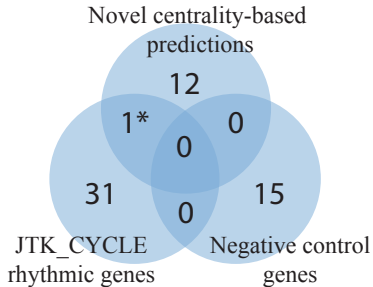

\* p value: N/A

B.

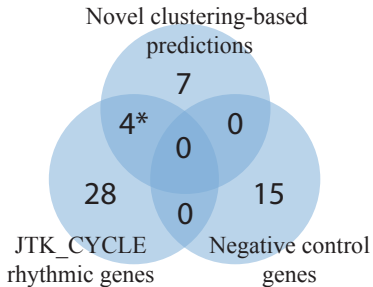

\* p value: 0.0064

Supplement: Additional file 7: — Validation of our network-based rhythmic genes in terms of GO term overlaps with positive and negative control genes. Pairwise overlaps of enriched GO terms between the JTK_CYCLE-identified rhythmic genes, negative controls, and our novel predictions produced by (A) centrality analysis or (B) the clustering analysis. (PDF 347 kb) [file 12864_2016_2998_MOESM7_ESM.pdf]
