# Supplementary material for: Genome-wide profiling of 24 hr diel rhythmicity in the water flea, Daphnia pulex: network analysis reveals rhythmic gene expression and enhances functional gene annotation
Source: BMC Genomics. 2016 Aug 18;17:653. doi: 10.1186/s12864-016-2998-2 (PMC4991082; doi:10.1186/s12864-016-2998-2)
Supplement: Additional file 3: — Microarray validation using qRT-PCR. Microarray validation was performed on two rhythmic genes, a salivary C-type lectin and a β,β-carotene-15,15′-dioxygenase using qRT-PCR. The qRT-PCR gene expression is relative to Alpha tubulin. Day and night are indicated by the horizontal white/black bars. Primer sequences are provided. (PDF 271 kb) [file 12864_2016_2998_MOESM3_ESM.pdf]

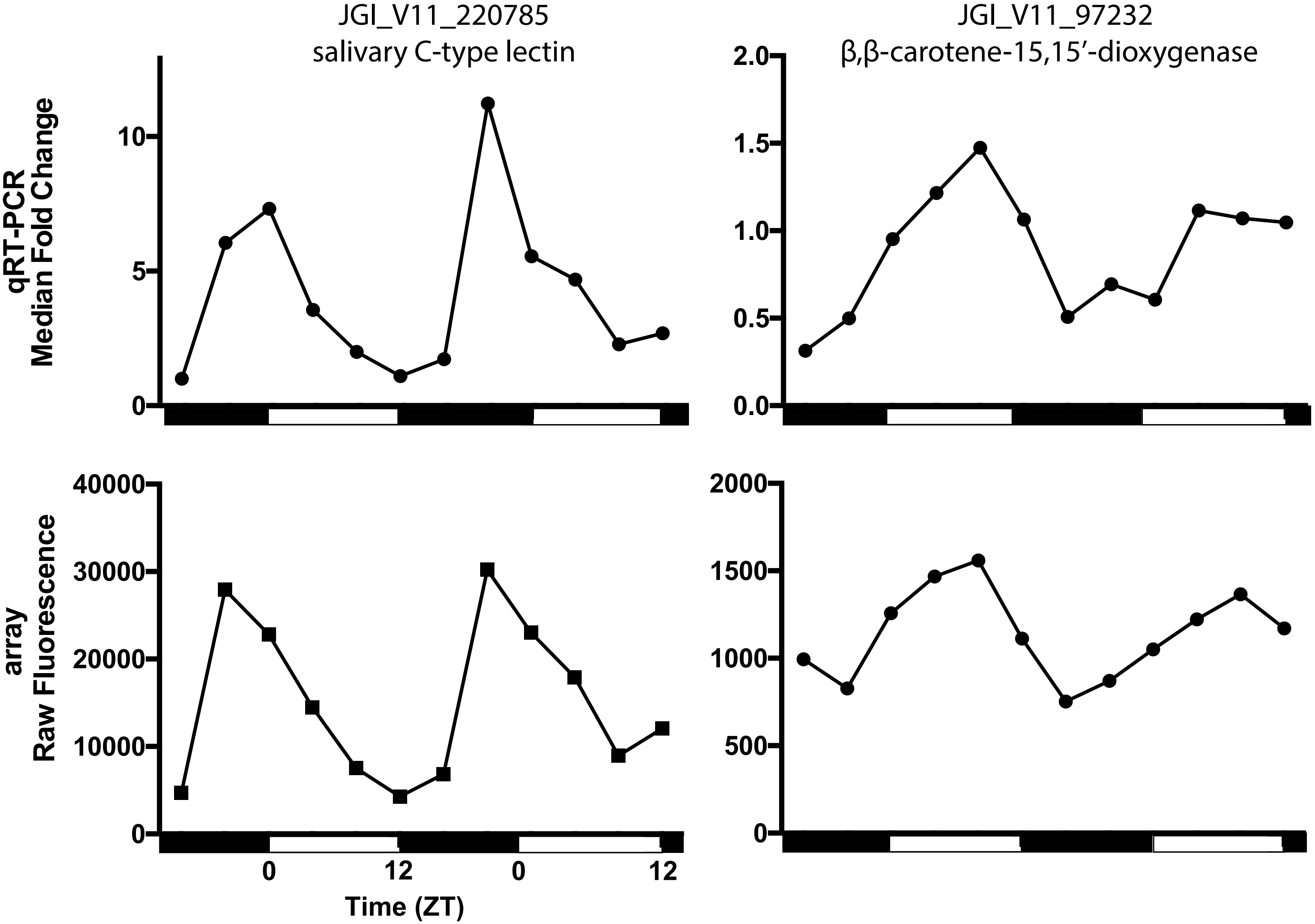

Gene	Accession no.	Orientation*	Primer sequence (5’ to 3’)
Alpha tubulin	JGI_V11_301837	F	CGATCTGATGTACGCCAAGC
		R	CTGCAAGATCTTCACGAGCC
salivary C-type lectin	JGI_V11_220785	F	AAACGGACCGAATTTTGGCA
		R	TTGGAGATTTTCGTCCCGGA
β,β-carotene-15,15’-dioxygenase	JGI_V11_97232	F	GCCAGAAAATCGGAGCCAAA
		R	TGCCTTGGACTCTTGATCGT

* F, forward; R, reverse
