# Supplementary material for: Genome-wide profiling of 24 hr diel rhythmicity in the water flea, Daphnia pulex: network analysis reveals rhythmic gene expression and enhances functional gene annotation
Source: BMC Genomics. 2016 Aug 18;17:653. doi: 10.1186/s12864-016-2998-2 (PMC4991082; doi:10.1186/s12864-016-2998-2)
Supplement: Additional file 4: — Properties of our networks. The size, density, average diameter, and average clustering coefficient of the five networks (i.e., their largest connected components), which we consider in our study. We also studied additional network properties, including the degree distribution, clustering spectrum, and graphlet frequencies (results not shown). (DOCX 57 kb) [file 12864_2016_2998_MOESM4_ESM.docx]

# Properties of our networks

|  | **Nodes** | **Edges** | **Density** | **Diameter** | **Clustering coefficient** |
| --- | --- | --- | --- | --- | --- |
| SIGN N | 3,383 | 22,955 | 0.40% | 6.48 | 0.26 |
| ABS N | 4,892 | 27,651 | 0.23% | 8.47 | 0.26 |
| MI N | 2,093 | 32,658 | 1.49% | 2.76 | 0.74 |
| ABS-MI-10N | 2,973 | 18,115 | 0.41% | 6.63 | 0.40 |
| ABS-MI-25N | 4,172 | 40,921 | 0.47% | 5.80 | 0.43 |

The size, density, average diameter, and average clustering coefficient of the five networks (*i.e.* their largest connected components), which we consider in our study. We also studied additional network properties, including the degree distribution, clustering spectrum, and graphlet frequencies (results not shown).
