# Supplementary material for: Genome-wide profiling of 24 hr diel rhythmicity in the water flea, Daphnia pulex: network analysis reveals rhythmic gene expression and enhances functional gene annotation
Source: BMC Genomics. 2016 Aug 18;17:653. doi: 10.1186/s12864-016-2998-2 (PMC4991082; doi:10.1186/s12864-016-2998-2)
Supplement: Additional file 5: — Intersections between our networks. Pairwise network intersections, in terms of the number of edges common to two given networks divided by the number of edges present in the union of the two networks. (DOCX 55 kb) [file 12864_2016_2998_MOESM5_ESM.docx]

# Intersections between our networks

|  | **SIGN N** | **ABS N** | **MI N** | **ABS-MI-10N** | **ABS-MI-25N** |
| --- | --- | --- | --- | --- | --- |
| SIGN N | 100.0% | 72.2% | 0.6% | 4.6% | 3.1% |
| ABS N | 72.2% | 100.0% | 0.9% | 6.2% | 4.4% |
| MI N | 0.6% | 0.9% | 100.0% | 5.3% | 6.5% |
| ABS-MI-10N | 4.6% | 6.2% | 5.3% | 100.0% | 44.3% |
| ABS-MI-25N | 3.1% | 4.4% | 6.5% | 44.3% | 100.0% |

Pairwise network intersections, in terms of the number of edges common to two given networks divided by the number of edges present in the union of the two networks.
