# Supplementary material for: Genome-wide profiling of 24 hr diel rhythmicity in the water flea, Daphnia pulex: network analysis reveals rhythmic gene expression and enhances functional gene annotation
Source: BMC Genomics. 2016 Aug 18;17:653. doi: 10.1186/s12864-016-2998-2 (PMC4991082; doi:10.1186/s12864-016-2998-2)
Supplement: Additional file 6: — Information on the rhythmic genes in our networks. The number of all nodes or the subset of the 1,661 JTK_CYCLE-identified rhythmic genes, for each network and all five networks combined. (DOCX 68 kb) [file 12864_2016_2998_MOESM6_ESM.docx]

# Information on the rhythmic genes in our networks

|  | **SIGN N** | **ABS N** | **MI N** | **ABS-MI-10N** | **ABS-MI-25N** | **All networks combined** |
| --- | --- | --- | --- | --- | --- | --- |
| Nodes | 3,383 | 4,892 | 2,093 | 2,973 | 4,172 | 7,262 |
| Rhythmic genes | 28 | 325 | 338 | 351 | 458 | 626 |

The number of all nodes or the subset of the 1,661 JTK_CYCLE-identified rhythmic genes, for each network and all five networks combined.
